# Abnormal mosaic flow in the left atrium observed from a parasternal long-axis view in a patient with a history of pulmonary vein isolation: a case report

**DOI:** 10.1093/ehjcr/ytad067

**Published:** 2023-02-07

**Authors:** Hironori Ishiguchi, Yasuhiro Yoshiga, Takayuki Okamura, Masafumi Yano

**Affiliations:** Division of Cardiology, Department of Medicine and Clinical Science, Yamaguchi University Graduate School of Medicine, 1-1-1 Minamikogushi, Ube, Yamaguchi 755-8505, Japan; Division of Cardiology, Department of Medicine and Clinical Science, Yamaguchi University Graduate School of Medicine, 1-1-1 Minamikogushi, Ube, Yamaguchi 755-8505, Japan; Division of Cardiology, Department of Medicine and Clinical Science, Yamaguchi University Graduate School of Medicine, 1-1-1 Minamikogushi, Ube, Yamaguchi 755-8505, Japan; Division of Cardiology, Department of Medicine and Clinical Science, Yamaguchi University Graduate School of Medicine, 1-1-1 Minamikogushi, Ube, Yamaguchi 755-8505, Japan

A 48-year-old male with a history of atrial fibrillation underwent radiofrequency pulmonary vein (PV) isolation. The patient had normal PVs (*Figure 1A*). First-pass and ipsilateral isolation were uneventfully accomplished in both the right and left PVs. Three months later, the patient was asymptomatic, had a normal chest radiograph (*Figure 1B*), and subsequently underwent echocardiography for regular follow-up. An abnormal mosaic flow in the left atrium, which was undetected preoperatively, was obtained from a parasternal long-axis view (*Figure 1C, arrow*, see Supplementary material online, *Movies S1* and *S2*). It was identified as accelerated PV flow on Doppler echocardiography (*Figure 1D*).

**Figure 1:**
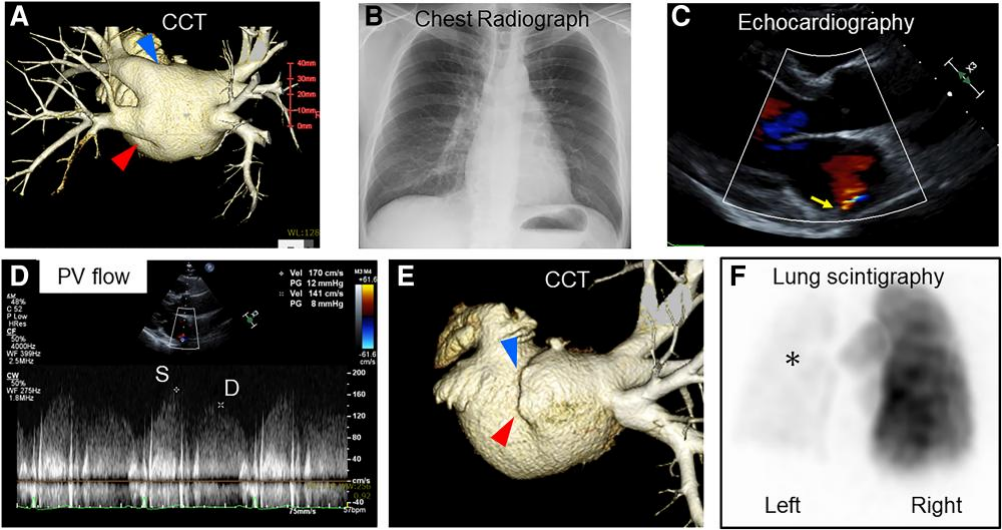
(*A*) Cardiac computed tomography image in postero-anterior view obtained before pulmonary vein isolation. Pulmonary veins were intact. Arrowheads indicate left superior and left inferior pulmonary veins, respectively. (*B*) Chest radiograph at 3 months after pulmonary vein isolation. (*C*) A mosaic flow in the left atrium from a parasternal long-axis view during echocardiography (arrow). (*D*) The continuous Doppler waveforms of pulmonary vein flow. The velocity of systolic (S) and diastolic (D) waves was 1.7 and 1.4 m/s, respectively. (*E*) Cardiac computed tomography in postero-anterior view detects pulmonary vein stenosis (arrowheads: ostium of the left superior- and left inferior pulmonary vein). (*F*) Lung scintigraphy with ^99m^Tc-macro-aggregated albumin in postero-anterior view indicating severe perfusion impairment of the left lung (asterisk). CCT, cardiac computed tomography; PV, pulmonary vein.

Cardiac computed tomography (CCT) was scheduled because PV stenosis was suspected. Cardiac computed tomography confirmed PV stenosis of both the left superior and inferior sides (*Figure 1E*). Lung scintigraphy showed severe perfusion impairment of the left lung (*Figure 1F*) with normal ventilation. The patient underwent implantation of a stent for the left superior PV (Express LD, Boston Scientific, Marlborough, MA, USA) and left inferior PV (Express SD), respectively. Postprocedural echocardiography revealed that the mosaic flow was replaced by the flow from the stent implanted in the left inferior PV (see Supplementary material online, *Movie S3*).

Although PV stenosis is a rare complication (with as low as 0.7% incidence), it remains a critical complication of PV isolation.^1–3^ Early detection and intervention before revascularization becomes challenging are warranted to treat the complication.^2,3^ In this case, PV stenosis was diagnosed early before the patient developed symptoms due to the identification of accelerated PV flow during echocardiography at a regular check-up.

Our case is educative because it highlights that clinicians should be cautious of accelerated flow in the left atrium when performing echocardiography for patients with a history of PV isolation, even if they are asymptomatic.

## Supplementary Material

ytad067_Supplementary_Data

## Supplementary material

**Consent:** The authors confirm that written consent for submission and publication of this case report including images and associated text has been obtained from the patient in line with COPE guidance.

**Funding :** None declared.
